# Development and experiences of an internet-based acceptance and commitment training (I-ACT) intervention in ice hockey players: a qualitative feasibility study

**DOI:** 10.3389/fspor.2024.1297631

**Published:** 2024-03-22

**Authors:** Gustaf Reinebo, Linda G. Björverud, Thomas Parling, Gerhard Andersson, Markus Jansson-Fröjmark, Tobias Lundgren

**Affiliations:** ^1^ Centre for Psychiatry Research, Department of Clinical Neuroscience, Karolinska Institutet, & Stockholm Health Care Services, Region Stockholm, Stockholm, Sweden; ^2^ Department of Behavioural Sciences and Learning, Department of Biomedical and Clinical Sciences, Linköping University, Linköping, Sweden

**Keywords:** internet-based intervention, acceptance and commitment therapy, sport psychology, ice hockey, qualitative research

## Abstract

Internet-based psychological interventions have increased the accessibility of evidence-based treatments in clinical psychology but are still an unexplored delivery format in sport psychology research. This study describes the development and evaluates the experiences of an internet-based acceptance and commitment therapy/training (I-ACT) intervention in ice hockey players focusing on performance enhancement and a sustainable sport participation. I-ACT consisted of seven weekly modules and the feasibility of the intervention was investigated using a qualitative research design. Four national level ice hockey players took part of I-ACT and were interviewed about their experiences using a semi-structured protocol. Interview transcripts were analyzed using qualitative content analysis. Findings suggest that the content of I-ACT was comprehensible, relevant, and that it was possible to put the psychological skills into practice. I-ACT was described as helpful to the ice hockey players either in their sport performance or in their life outside of sport. The internet-format was generally perceived as positive, flexible, and a feasible option for delivering psychological interventions in an elite sport context. Some concerns were raised regarding the timing of the intervention at the end of the season, and some players also wished for more time to complete I-ACT. It was also expressed that some of the exercises could have been better adapted for goaltenders. Further trials are needed to evaluate the effects of I-ACT on performance and mental health outcomes in various sport populations using robust quantitative research methodology. Internet-based psychological interventions are a potential future opportunity to make evidence-based practices more accessible for athletes.

## Introduction

Internet-based psychological interventions have the potential to make evidence-based psychological interventions more accessible and reach more people in need. This is evident in clinical psychology, where internet-delivered interventions have created new innovative ways of reaching and treating humans suffering from psychiatric conditions (1). The internet intervention can either be self-guided or administered with an added possibility to communicate by text with a therapist (often called “therapist-guided”). The internet therapist can either answer questions and support the participant, or direct the participant to relevant material/exercises in the intervention. Therapist-guided internet interventions have shown superior results to self-guided interventions (2, 3). Internet-based cognitive behavior therapy (I-CBT), the therapy form most studied so far, has shown equal results to traditional face-to-face CBT for depression and anxiety disorders (4, 5). In some studies, it has also been shown to be favorable from a cost-effectiveness perspective (6). Thus, further exploration of the internet-based psychological intervention format into new applied areas is warranted for several reasons.

Sport psychology consultancy is a common practice worldwide and the potential of using internet-based interventions in sports is to a large degree still unexplored territory. Starting about a decade ago, two master theses investigated the effects of internet-based CBT techniques on psychological skills in athletes. Randomized controlled designs were used and one study included 70 coactive athletes (7), while the other study included 44 athletes (8). Competitive anxiety, perceived stress, mindful awareness, and psychological skills were investigated in both studies. The first study showed no significant effects although positive attitudes towards the training were reported qualitatively (7). The second study showed significant effects on psychological skills (8). Although the potential of internet-based interventions was acknowledged around this time (9), the number of published studies was non-existent during the following years (10, 11). However, online interventions have just recently started to be applied and evaluated in sport psychology. A mindfulness intervention was compared to a self-regulation intervention in middle-distance runners and evaluated with qualitative methods (12). The purpose of the interventions was to impact exercise-induced pain, mental performance, and mental health. The qualitative analysis, using a codebook thematic analysis approach, indicated that both interventions helped the athletes in all these respects—with their exercise-induced pain, mental health, and mental performance. In another study, a three-armed randomized trial compared self-talk, implementation intention strategies, and an inactive control in recreational endurance athletes (13). The two interventions were brief (10–15 min) and designed to help the athletes in their performance. Subjective performance, goal attainment, coping strategies, perceived stress, and self-efficacy were used as dependent variables. A significant effect was found on stress controllability for the implementation intention group in comparison to the inactive control group. Lastly, a blended intervention (mix of online and face-to-face intervention) based on acceptance and commitment therapy (ACT) was evaluated in a sample of female soccer players (14). The aim of the intervention was to improve mental well-being and to help the athletes face various struggles both related to their sport and in life outside sports. Three face-to-face group sessions were followed by a six-week online intervention. In the ACT group, psychological flexibility, as measured with the Psychological Flexibility in Sport Scale (PFSS) (15) increased, while depression and stress symptoms declined. Further, social and emotional well-being was maintained compared to the control group. These studies are part of the recent innovative exploration of applying internet-based psychological interventions in sports and have a potential ability to increase the availability of psychological services to aid athletes. However, there is yet no study describing the development of an internet-based psychological intervention in the context of sport and investigating the experiences of the internet-based format.

ACT is a contextual behavioral change model and is developed from cognitive and behavior therapy (16). ACT is often described as being part of the third wave of cognitive and behavior therapies, referring to a development from CBT. ACTs theoretical foundation is applied behavioral analysis and a specific theory of language and cognition called Relational Frame Theory (RFT) (17). ACT was primarily applied and tested in clinical psychology and has been shown to successfully treat a number of psychiatric and somatic conditions (18). ACT has also been adapted and evaluated in other areas, including sports (19–21). The aim of ACT is to foster psychological flexibility (PF). PF is defined as the ability to fully contact the present moment and the thoughts and feelings it contains without needless defense, and, depending upon what the situation affords, persisting in or changing behavior in the pursuit of goals and values (22). PF is developed by applying interventions that target the central processes of change in the ACT model; acceptance, defusion, self-as-context, present moment awareness, values, and committed action (23). In short, acceptance refers to the ongoing willingness to embrace inner experiences as they present themselves in given situations without trying to change their frequency or form. Defusion is to treat thoughts as thoughts and not as literal truth and by doing so creating a space for the athlete to behave efficiently towards what's important in the moment. Practicing present moment awareness train the athlete's ability to consciously direct their attention to chosen stimuli (inner or outer experiences) in a non-judgmental way, and self-as-context is the ongoing process of noticing these experiences as separate from the self. Values are what is important and meaningful for the athlete and can be regarding both the life outside of sports or regarding the sporting activity itself. Committed actions are actions that reflect the athlete's chosen values on how they want to approach their sporting activity, performance and life outside sports, and is another behavioral process in ACT (20, 23, 24). When psychological interventions are implemented in sports to enhance performance, the primary aim is different from the aim of treating psychiatric conditions. For instance, when the population is not primarily psychiatric it can focus on enhancing performance and/or promoting a healthy and sustainable participation in sport that focuses on processes of well-being from a psychological perspective (19, 25). When ACT is used as a psychological training method, e.g., to enhance performance rather than treat psychiatric conditions, sometimes “therapy” is replaced with “training” in the ACT-acronym. The method has also heavily influenced other variants of mindfulness and acceptance-based approaches that have been developed during the last two decades in sports (11, 26). One sport population where ACT has been evaluated is ice hockey. A four-session ACT intervention delivered in a group format increased ice hockey-related psychological flexibility compared to a control group (20). A subsequent randomized trial found effects on objective performance outcomes as measured with goals, assists and taken shots. Blinded coach ratings of players performance, focus, and commitment of their development were also found to be superior in the ACT group compared to the control group (19). The overarching aim of this study is to further investigate ACT in sports, and in ice hockey specifically, by evaluating the feasibility of using an internet-based format for delivering the intervention.

### Purpose and aim of the study

The purpose of this study is to describe the development and evaluate the feasibility and experiences of an internet-based psychological intervention in sports. The intervention will be based on acceptance and commitment therapy/training (ACT) focusing on performance enhancement and a sustainable sport participation. The internet-based ACT intervention (I-ACT) will be tested in ice hockey players.

#### Research questions

This study sought to investigate the following research questions among ice-hockey players:

(1) Was the content in I-ACT comprehensible and relevant? Was it adapted to their needs?
(2) How did the participants experience the internet-based format and the various mediums of I-ACT (text, audio, video)?
(3) What was the experience of the I-ACT content and was the scope of the intervention feasible and possible to combine with their other training?
(4) Was it possible for the participants to convert the psychological skills from I-ACT into practice?

## Method

### Philosophical assumptions

A contextual behavioral approach has been applied throughout all stages of this study and has informed both the intervention content and analyses. The philosophy that underpins contextual behavioral science (CBS) (27) is a version of contextualism known as functional contextualism (28–30). The epistemology of functional contextualism is derived from behavioral pragmatism (28, 31), and its goal is to predict and influence behavior with precision, scope, and depth (32). The root metaphor and unit of analysis in functional contextualism is the ongoing act-in-context, which is the behavior of a whole organism considered in and inseparable from its situational and historical context. The function (i.e., understanding) of behavior emerges through the relationships of the various parts in the analysis as a whole (the behavior in context), as the separate parts of the analysis have no meaning by themselves (33). This study constitutes both the development and adaptation of psychological intervention material and an analysis of the experiences from its implementation, for which a qualitative research methodology has been adopted.

### Ethical considerations

This research was ethically approved by the Swedish ethics review authority (registration number 2019-01175). All participants received information about the research and gave their digital consent prior to taking part of the internet-based psychological intervention. They also returned their written consent at the time of the semi-structured interview.

### Procedure

#### Development of I-ACT

The initial phase of the study was the development of the internet-based acceptance and commitment therapy (I-ACT) program. This version of I-ACT was developed for ice hockey players which in practice meant that the examples outlined in the intervention were sport specific. Content from the protocol described in Lundgren et al. (20) was used as an outline for I-ACT. The ACT intervention in Lundgren et al. (20) was a four session group intervention and included exercises such as the Sport lifeline, the Bull's Eye, the juggler and your bag in life. I-ACT includes these exercises in internet-based form but also other ACT related material. The first author (GR) was the main developer of the I-ACT program content, and feedback was provided by other authors during the development process. All content was in the Swedish language. Taken together, the author team consist of psychologists, psychotherapists and researchers trained in cognitive behavior therapy (CBT) and ACT applied in both clinical and sports contexts. I-ACT consists of seven modules with material and therapeutic exercises in text, video and audio that the participant interacts with to train ACT skills. Participants write down their own experiences in relation to the exercises and are given homework assignments to help implement the skills in their sport and life. Some text sections or videos were designed to be informative and educative, but other material such as audio exercises in mindfulness and other guided ACT exercises were designed to promote experiential training in ACT skills (e.g., the leaves in the river exercise in module 3 about defusion). Further examples of how exercises could be designed was the Sport life line (34) and the Sports Bulls Eye (35) that was text-based. In the Sports Bulls Eye for example, values and values-based behavior in sport are first described. Then the athlete identifies their own values in important areas for their athletic endeavor and rate to what extent they behave in line with this. They also identify possible obstacles and form a committed action plan. Participants could write down their experiences and thoughts in text boxes on the web platform in relation to the exercise. Another example of a video exercise design (e.g., The juggler exercise in module 3 or The bag in your life exercise in module 4) were role plays that was modeled with the first (GR) and last author (TL) to demonstrate acceptance and defusion skills in a similar way that would have been done in a face-to-face session. After seeing the videos, the participants were asked to reflect on what they had seen, write down what thoughts and emotions they would have brought up if they were the client in the session, and how they could train on the ACT skills in their own life and sport. Modules were designed so that participants would take part of ACT material in a stepwise fashion. The first module introduces the program and focuses on values work and committed action and sets up the behavioral plan for the intervention. Mindfulness and awareness in sport is introduced in module 2, and thereafter defusion (module 3) and acceptance skills (module 4) while the participant is also introduced to behavior analytic concepts to better understand the behaviors they are trying to change and improve using I-ACT. Module 5 focuses on putting the skills together, handling pressure, sleep and diet routines, and other behavioral and psychological aspects important to the athletic endeavor. Module 6 focuses on handling setbacks and also dives more into values and committed action plan for the life outside sports to promote balance in life. Module 7 focuses on repetition and also helps the athlete to form a maintenance plan to keep working on the ACT skills. The final content of I-ACT is described in Supplemenatry Table S1.

#### Participants

Four male ice hockey players, from the same club, were recruited from Hockeyallsvenskan (the second highest league in Swedish ice hockey for men) to take part of I-ACT. Initial contact was made with the club, and these four players volunteered to participate in the research project. Their positions were as follows: two forwards, one defender, and one goaltender. Participants were screened for mental health issues, and, based on results on measures for depression (the Patient Health Questionnaire, PHQ-9) M = 5, SD = 2.58 (36), stress (Perceived stress scale, PSS-10) M = 16.5, SD = 3.87 (37), and general anxiety (Generalized anxiety disorder, GAD-7) M = 5.25, SD = 1.71 (38), the sample was considered non-clinical. The players were interested in taking part in a psychological intervention to enhance their performance. They were all fluent in the Swedish language. The mean age was 26 years (SD = 1.63), and the time playing at the current or higher performance level was 2.75 years (SD = 2.87). When the players registered for the research project on the web-platform they also answered questions about their previous experiences with sport psychology consultancy. Two of the players had no previous experience of sport psychologist consultancy/interventions. One player had previous experience of working with a sport psychologist for more than two years ago and stated positive experiences. Another player had previous experience of working with a sport psychologist for more than one year ago and stated both positive and negative experiences.

#### Implementation of I-ACT

The four ice hockey players took part of I-ACT during the spring of 2019 (March-May). The seven modules of I-ACT were administered weekly. As this was during the final period of the season, some parts of I-ACT were administered off-season. I-ACT was implemented and delivered from a web platform called “Iterapi” which has been used for multiple previous studies on internet-based interventions in clinical psychology (39). Please see Vlaescu et al. (39) for a thorough description of the platform's functionality and pictures of it. I-ACT was administered as a therapist-guided internet intervention which meant that the participant could be assisted by a therapist via a chat function on the web platform. The first author (GR), who is a licensed psychologist with a background in clinical psychology and is currently a PhD candidate in sport psychology, was the internet therapist for all participants in this study. The therapist sent a chat message once a week to check in on how it was going with I-ACT if the participants had not already contacted the therapist during that particular week. The players rated various aspects of the I-ACT material after each module to evaluate the content of the intervention.

#### Semi-structured interviews

After completing I-ACT, semi-structured interviews were conducted with all four ice hockey players to evaluate their experiences of the intervention. The interviews were conducted in June 2019 by the first author (GR) at the training facilities of the players club. Mean time of the interviews was 72.25 min (SD = 12.53). The semi-structured interview protocol can be provided by the corresponding author (GR) upon request.

### Qualitative analysis

Qualitative content analysis with an inductive approach was used to analyze the transcripts from the semi-structured interviews (40). It is a method that emphasizes variation and identifies patterns of both similarities and differences in the text. It focuses on both the subject and the context and can contain varying levels of interpretations of the data, both manifest (close to the text) and latent (interpretative and inferred) (41). In the current study, the research questions had the potential to correspond to varying interpretative levels of the data, both semantic (e.g., describing what type of device they used when working with I-ACT) and to some degree inferred (e.g., describing a therapeutic experience of the program and its meaning for the athlete). Therefore, the content analysis involved a combination of a codebook approach with a varying level of interpretation (42). The qualitative content analysis process starts with identifying meaningful units of information (called codes) in the text that corresponds to the research questions. These codes are sorted into sub-categories and categories (also known as “abstraction”) that organize the data into patterns and themes of information and knowledge found in the text (40, 42). Two of the authors (GR and LB) read through all transcripts and identified meaningful units of information and coded all material separately. Then, the two authors assessed all meaningful units of information with respective codes together and reached consensus through discussion. The first author (GR) abstracted sub-categories, categories, and descriptive themes from the final codes (43). Microsoft Excel (Microsoft 365, Version 16.81) was used to organize the abstraction. LB then calibrated the abstraction of sub-categories, categories, and themes. This process was also calibrated and discussed with the last author (TL). The raw text and codes were continuously revisited through all stages of analysis.

## Results

### Qualitative content analysis

A total of 445 meaningful text units were identified and coded from the four interviews that corresponded to the research questions. These were sorted into 34 sub-categories, 7 categories, and 2 themes (see Supplemenatry Table S2). The two themes were (A) The development of and challenges with an internet-based psychological intervention for athletes, and (B) The relevance of psychological skills. The seven categories and associated sub-categories are described in the text below.

### Categories of theme A: the development of and challenges with an internet-based psychological intervention for athletes

#### The internet-format

The ice hockey players expressed their views on how it was to engage in a digital psychological intervention. The participants thought the internet format made the psychological training accessible. They could access the intervention with their phone at any time, everything was available in one and the same place. The internet format was flexible in the sense that participants could engage in the intervention whenever it suited them.

“An advantage is that I have access to it all the time.” (P1)

It was also said that psychological support can be hard to find, and that the internet format is an alternative to reach more athletes. All players expressed their appreciation for the internet format in general and two of them expressed that it was better than expected.

“It |the internet format] was over my expectations! Although I didn’t really know what to expect. But it was much easier than I thought.” (P3)

Most players used I-ACT on their phones and commonly worked with it from home, however, one player also said he used it on the bus during away games. The internet format enabled the players to focus more on the material they thought was the most rewarding parts—which varied between them. The internet format made it clear that the responsibility of engaging in psychological training is up to the athlete. Three of the players had partial concerns regarding the internet format. To always have the accessibility to the intervention can also be “too much” availability, and potential response latency from the sport psychology consultant could be negative. Further, writing down and formulating struggles could be difficult, text communication has a potentially higher risk of being misunderstood, and to do audio exercises at home alone could be difficult according to one player.

#### Pedagogics

Overall, the participants expressed that the I-ACT content was understandable and easy to absorb. The digital format gave some pedagogic advantages, such as the possibility to go back and repeat parts of the material and two of the players expressed it was a positive experience to write down their struggles. Furthermore, all athletes thought it was positive that the intervention was delivered in different media formats (text, audio exercises, and videos). Different formats complemented each other which was good for intervention comprehensibility and the learning process.

“If you look at a picture or listen to audio [exercises], you understand it better. You perceive it better and understand it in a completely different way when it’s delivered as audio, pictures or video.” … “I thought all three [media formats] were necessary. Some videos and audio exercises sort of stuck with me.” (P4)

Two of the players expressed that I-ACT had a good structure and one said it was helpful with specific hockey examples. Participants showed ACT consistent understanding when describing what the intervention gave them, such as the importance of acceptance towards inner experiences, present moment awareness and to challenge personal fears.

#### Improvement areas of I-ACT

In comparison with other formats, two athletes expressed that talking face-to-face has its advantages, such as being a more efficient or a deeper way of communicating, and that it could serve as a complement to the internet-based format. One of the two players would have liked phone calls as a supplement to I-ACT during the intervention.

“…maybe talk on the phone sometime during the modules.” …. “I’m not sure but maybe it gets a little deeper if you talk than if you write but yeah, I mean we are made to talk right.” (P4)

Concerns were also raised regarding the timing of the intervention being at the end of the season and even post-season, which some of them considered non-optimal. Two of the players wished for more time to complete the I-ACT program. One player expressed some concerns regarding the amount of text to read and wished for more videos. He also described difficulties understanding the juggling-exercise (defusion) and he also found the implementation of the substitution-box-routine stressful at first (specifically the part of noticing the breath), although he later adapted the routine in a way that worked better for him. The goaltender thought that some of the exercises were too focused on outfield players and could have been better adapted for goaltenders.

#### Procedure feasibility

All players said it was positive and helpful with the chat function with the psychologist and that quick answers were provided when needed. It was also good that the psychologist contacted them on a regular basis to check-in on how they were doing, and that once a week was a reasonable level.

“I know I wrote to you after a game. We faced [a team] and I was part of the conceded goal that cost us the game … a thing like that, to just write to someone to let go of it”. (P3)

In general, they also thought that the amount of content in I-ACT and the number of questionnaires was reasonable.

#### The role of the context during an individual psychological intervention in sports

There were other players in the team that did not take part in I-ACT who knew that some players did, and there was a positive sense of curiosity from some of the players who did not take part. Two of the participating players encouraged each other during I-ACT with reminders to try again in what was challenging for them.

“It was mostly about reminding each other about things [two of the I-ACT participating players]. That’s pretty much it. It’s not like we have said what we have written to you [the psychologist] or something like that. But maybe more about game situations, like maybe asking what did you think I did well today? It has been on that level. And then during hockey training sessions helping each other to dare trying all the time. Yes well if it doesn’t work then ahh damn it let’s go again, try again. A bit like that.” (P3)

One player did not think that the type of club culture would have mattered for taking part in I-ACT. Another player said that the positive climate in the current club was open and allowing which was good circumstances for taking part of I-ACT. However, he also said that the positive climate already brought some psychological support and that the need for I-ACT could have been greater for the individual if that would not have been the case. One player said that it could have been positive to include coach feedback on the sport-specific parts of the work with I-ACT.

### Categories of theme B: the relevance of psychological skills

#### It was possible to put the skills into practice

All players expressed that the psychological skills that were trained in the program could be implemented with their sport behavior. Further, examples were given that described different kinds of ACT coherent behaviors, such as letting go of thoughts, staying present, redirecting their focus on what's coming next, challenging fears, and making room for mistakes to improve in the long run.

"… this about seeing the opportunities and not trying to avoid, to face your fear instead of trying to avoid it.” (P2)

All players gave examples of exercises in I-ACT that they thought had been useful for implementing psychological skills in their hockey game and performance. Some examples were the Match Bulls-Eye, the Sports Lifeline, and the substitution-box-routine.

“One main focus [using Match Bulls-Eye] that I could bring on to the ice. It was then easy for me to focus on….I also used Match Bulls-Eye during training session to remember what to practice on …” (P1)

Other examples of exercises described as rewarding were guided mindfulness and other audio exercises, as well as acceptance- and defusion-related content. One player thought that the sleep hygiene and diet material was helpful, and another said that the sport-closure-exercise was helpful. The players also described that the psychological skills could be automated and informally trained into their sport behavior over time, and it was common to focus on certain helpful exercises rather than the formal homework assignments that were given at the end of each module. Further, they also said that the psychological skills required practice to be established in their behavioral repertoire. Examples of skills being generalized to other activities were also given, such as being applied during strength conditioning training according to two of the players.

#### I-ACT was helpful

All players expressed that I-ACT met their needs and was relevant to them as ice hockey players. The content was experienced as broad and possible to tailor to their individual needs, and they thought that other players would benefit from the psychological intervention as well. All players gave examples on how the psychological intervention was helpful to them in relation to their game and as athletes. It could, for example, be about handling negative thoughts and emotions better, improved focus, to be more confident or relaxed during performance, challenging oneself more during performance, or finding a more consistent performance level. Examples were also given that I-ACT could help to create more efficient behaviors and routines that saved time and energy.

“It was about finding your way back to your game all the time. To go from having a good period, and then have a dip during the season and finding your way back. It took much longer before I started with this [I-ACT]. When I started with this it was more of a clap on your shoulder more often and there were no dips really. So it became much easier. I didn’t think it would be so rewarding as it was.” (P3)

All players said that I-ACT was helpful in life outside of sports. Some examples given were regarding relationships, improved ability to handle thoughts and emotions, improved sleep, or new perspectives on life and being better at separating between the sport and the life outside sports.

“If there is anything at home or something with the family that maybe makes me a bit angry or something, I then felt that I started to apply these things and I could then like handle it better.” (P4)

All athletes were asked about negative effects of the intervention and although there were some challenges with the program (see category “Improvement areas of I-ACT”), none reported negative effects on their lives or athletic endeavor. In general, the players appreciated the psychological intervention and three of them even expressed it was better than expected.

### Content ratings of the I-ACT modules

The content ratings of the various aspects of the I-ACT modules are presented in Supplemenatry Table S3.

## Discussion

This study describes the development and evaluates the experiences of an internet-based ACT intervention in ice hockey players. I-ACT was designed to focus on performance enhancement and promoting a sustainable sport participation by practicing skills to increase psychological flexibility. Four ice hockey players competing on national level in Sweden took part of I-ACT and were interviewed about their experiences using a semi-structured interview protocol. The transcripts of the interviews were analyzed using qualitative content analysis and the players did also rate various aspects of the content in I-ACT after each module. The main results of the qualitative analyses indicate that the research questions were answered satisfactorily. The content of I-ACT was comprehensible and reported as relevant to the participants and that it met their needs and expectations. Experiences of the internet format were positive in general. The flexibility that an internet-based delivery implicates, and the various media formats of the intervention (text, audio, and video) were appreciated and strengthened the pedagogics of the program. The participants would recommend I-ACT to other ice hockey players, which is in line with previous research when evaluating the feasibility of applying ACT in ice hockey in a group intervention face-to-face format (20). The results were also supported by the quantitative content ratings completed by the players after each module in I-ACT. There were some concerns regarding the timing of the intervention at the end of the season and even post-season. Some players also wished for more time to complete I-ACT. The participating goaltender expressed that some of the exercises were too focused on outfield players and could have been better adapted for goaltenders. Participants completed most of the seven modules (see Supplemenatry Table S3) which indicates that it was also feasible to get through most of the intervention material. It was also reported that the psychological skills of I-ACT could be put into practice and be of help either in their sport or in their life outside of sports. The results also indicate that minor adjustments can be made in upcoming trials (e.g., to further tailoring some of the intervention exercises to be better adapted for goaltenders, or the timing of when to implement the intervention during the season). Overall, the result of this study suggests that I-ACT is a feasible intervention in a sport context and would benefit from further experimental investigations of its quantitative effects on relevant sport and psychological outcomes.

The current study adds to the recent and growing literature which suggests that internet-based psychological interventions is a feasible delivery option for athletes in need of different kinds of psychological support (12–14). This study brings something new to the sport psychology literature by thoroughly outlining how an internet-based psychological intervention for athletes can be developed and evaluated for its relevancy and feasibility prior to testing its efficacy. In comparison to clinical psychology where internet-based interventions have been researched most extensively so far, the evidence for psychological interventions in sports, both over the internet and face-to-face, is far behind. However, internet-based interventions hold the same opportunity as a “vehicle of innovation” for making evidence-based interventions more accessible in sports, as it has in traditional clinical psychology (1).

### Strengths and limitations

The primary aim of this study was to describe the development and evaluate the experiences of an internet-based ACT intervention in ice hockey players. The results of this study cannot draw any conclusions about the efficacy for e.g., enhancing sport performance, since the research methods applied, or measures used, are not adequate to make such claims. To further evaluate the efficacy of I-ACT for athletes, research methods that are more adequate for drawing causal inferences should be used. Either in group-based format such as a randomized trial or using a single-subject experimental design (44).

A limitation of this study was that no female athletes were included. This limits the perspectives and experiences in the data that should be as multi-faceted as possible. Another aspect of the sample is that ice hockey was the only studied sport. The generalizability outside of ice hockey is therefore unknown. There are suggestions that sport specific adapted interventions can be beneficial and easier to apply and relate to sport-specific behaviors (45). Therefore, this version of I-ACT was adapted with ice hockey specific examples. This research group also has experience from conducting research and applying ACT in ice hockey, which has shown to be both feasible and an efficacious method when applied face-to-face (19, 20). A methodology concept often discussed in qualitative research is the saturation of data. The sample in this study consisted of four male ice hockey players competing on national level in Sweden, and the findings of this study should be interpreted acknowledging the limitations of this. However, the concept of saturation has also been questioned as a valid and useful concept in qualitative research, and more importantly, data should be assessed from the perspective of how well it can answer the specific research questions of the particular study (46). The aim and specificity of the research is therefore paramount when sample size in a qualitative design is to be considered “small” or “large” enough (47). The results in the current study suggest that internet-based ACT indeed was feasible and that the content was considered relevant for elite ice hockey players. This was also the primary aim to explore in this study.

It should be acknowledged from a methodological perspective that one and the same person (the first author, GR) was the main developer of the internet-based intervention, the therapist for the players, and the one who conducted the interviews. Although other authors were part of these processes as well, it cannot be ruled out to have biased the data collection to some degree. For instance, by letting the therapist interview his own clients about their experiences of their work together and the intervention that they knew the therapist was part of developing, can likely be influenced by socially conditioned responses. At the same time, no one else had a better grasp of the intervention or the individual client intervention processes, which meant that by having a thorough pre-understanding, detailed follow-up questions could be asked during the interviews. The role of the first author (GR) in the project described above should also be acknowledged regarding the data analysis and how it may have influenced the reflexivity of the analysis process (48). For instance, it is not farfetched that there was a wish that the participants would like the intervention that was designed to help them. Such aspects should be taken into account when interpreting the results even if the intention was to analyze the results as neutrally as possible in such respects. As part of handling this, two authors qualitatively analyzed the data, first separately, and then also discussed the interpretations of the meanings of the data. No inter-rater reliability measure was used, however, such methods have been questioned as a valid assumption of scientific rigor in qualitative research (49). It was also important to gather objective data on how much of the intervention they completed and the subjective ratings of the intervention content as part of the feasibility evaluation, which strengthens the interpretability of the participantś experiences. The subjective content ratings are not free from potentially being influenced by socially conditioned responses to some degree but are not as direct as an interview.

### Future research directions

Internet-based ACT has shown to be a feasible option for delivering an individual psychological intervention in an elite sport context. Future studies are needed to investigate the potential effects of I-ACT in athletes using robust research methodology that enable causal inferences about intervention effects, such as randomized trials or single-subject experimental design studies. The choices of outcome measures should also reflect the aim of the intervention. For instance, this version of I-ACT focused on performance enhancement and to promote a sustainable sports participation rather than treating psychiatric conditions. When this is the aim of future trials, it would be of interest to investigate the effects on both sport relevant (e.g., performance), psychological constructs related to well-being (e.g., life satisfaction, anxiety), and targeted psychological processes in the intervention (e.g., psychological flexibility) which can also be part of the research design to investigate mediating effects. I-ACT should also eventually be tested in further sports outside of ice hockey.

## Conclusions

This study describes the development and evaluates the experiences of an internet-based ACT intervention in ice hockey players. The internet-format was a feasible delivery option for providing psychological services in an elite sport context. The I-ACT content was comprehensible, relevant, and helpful to the ice hockey players, either in their sport performance or in their life outside of sport. Concerns were raised regarding the timing of the intervention which suggest that the intervention planning during the competitive season is important to consider. Internet-based psychological interventions are a promising alternative for increasing the accessibility to evidence-based interventions for athletes as it has in other areas of applied psychology. Further quantitative trials are needed to test the effects of I-ACT in various athlete populations using robust research methodology.

## Data Availability

The datasets presented in this article are not readily available because the original transcripts from the interviews contain information that could compromise the privacy of research participants. Only part of the data (abstracted codes in the qualitative content analysis) that support the findings of this study are available upon reasonable request from the corresponding author. Requests to access the datasets should be directed to gustaf.reinebo@ki.se.
