# Supplementary material for: Development and experiences of an internet-based acceptance and commitment training (I-ACT) intervention in ice hockey players: a qualitative feasibility study
Source: Front Sports Act Living. 2024 Mar 22;6:1297631. doi: 10.3389/fspor.2024.1297631 (PMC10995355; doi:10.3389/fspor.2024.1297631)
Supplement: Supplementary file 1 [file Table1.docx]

**Table 1.** I-ACT content

| Modules and main focus/ACT processes | Chapters & exercises  (All modules contain a homework assignment) |
| --- | --- |
| 1. The Sport lifeline - Values & committed action | Introduction  Sports Bulls Eye (values exercise)  The Sport lifeline (committed action & identifying goal behaviors)  Thoughts, feelings, behaviors (psychoeducation)  Motivation (psychoeducation) |
| 2. Refocusing - Mindfulness | Refocusing - to deliberately direct your attention  Mindfulness exercise (audio)  Focus and prepare for your performance (introducing the Match Bulls Eye exercise)  Substitution box routine and refocusing strategies  Evaluation and performance closure (completing and making a routine for the Match Bulls Eye exercise) |
| 3. Let the thoughts bounce - Defusion | Thinking - pros & cons (psychoeducation) Behavior analysis exercise  The breath staircase (audio mindfulness exercise)  The ice bear exercise (experiential/psychoeducation exercise to illustrate the problem with trying to control  your thoughts)  The juggler (video defusion exercise)  The leaves on the river (audio defusion exercise) |
| 4. Feelings and performance - Acceptance | Feelings and performance (psychoeducation) How can feelings affect your performance? (exercise: identifying sport situations & related emotional  responses) Behavior analysis exercise Audio mindfulness exercise (focusing on self-as-context) The polygraph (psychoeducation exercise to illustrate the problem with efforts for emotional control & avoidance) Your bag in life (video acceptance exercise) Exposure & safety behaviors |
| 5. Be a flexible ice hockey player - Putting the skills together | Revisit your Sports Bulls Eye (values exercise) Bold move (committed action exercise to challenge your fears and avoidance patterns) Find your pain (deepened reflection to connect bold moves, exposure and committed action behavior patterns) Big game players (reflective exercise on playing high-pressure games/situations) Professionalism (reflective exercise on how to approach various aspects of the athletic journey) Mindfulness audio exercise (body scan) I as a teammate (an ACT perspective on what characterize a functioning team including a reflective exercise on  how the athlete wants to approach being a teammate) Preparation & recovery (psychoeducation regarding important life routines including behavioral strategies for  sleep and diet) |
| 6. When things go to hell - Handle setbacks | Introduction and reflective exercise on previous setbacks and behavior strategies End of career exercise (audio values exercise) The letter (writing exercise focusing on values and perspective-taking based on the end-of-career-  exercise. The athlete writes a letter from a future to the present self on what is important and how to  approach their life as an athlete)  Life balance (psychoeducation about the importance of focusing on other aspects of life besides the sport) Your life compass (values exercise focusing on the life outside sport) Mindfulness audio exercise focusing on acceptance and self-as-context for memories of performance failures |
| 7. Maintenance plan: keep training and developing! | Repetition of central ACT skills (refocusing, defusion, & acceptance) My psychological training routine (creating an individual maintenance plan: summarizing learned skills,  identifying hinders for continue working with them, and how to get back to the psychological training in the  future if needed) Next step in my ice hockey (audio exercise focusing on the athletic journey so far and what the athlete would  want to come next. Values, committed action, and self-as-context processes) Bold move exercise focusing on next career steps (committed action exercise to challenge your fears and  avoidance patterns). Revisiting your Sports Bulls Eye (values exercise) |
