# Supplementary material for: Development and experiences of an internet-based acceptance and commitment training (I-ACT) intervention in ice hockey players: a qualitative feasibility study
Source: Front Sports Act Living. 2024 Mar 22;6:1297631. doi: 10.3389/fspor.2024.1297631 (PMC10995355; doi:10.3389/fspor.2024.1297631)
Supplement: Supplementary file 2 [file Table2.docx]

**Table 2.** Themes, categories and sub-categories

| **Themes** | **Categories** | **Sub-categories** |
| --- | --- | --- |
| The development of and challenges with an internet-based psychological intervention for athletes | The internet-format | The responsibility for the psychological training lies with the athlete |
|  |  | How I have used I-ACT |
|  |  | The internet format made psychological training accessible and flexible |
|  |  | The internet format was appreciated |
|  |  | Difficulties with the internet format |
|  | Pedagogics | ACT consistent understanding |
|  |  | The internet format had educational advantages |
|  |  | Appreciated educational elements |
|  |  | It was possible to understand I-ACT |
|  |  | Instructive |
|  |  | Educational that the intervention was delivered through various media (text, video, audio) |
|  | Improvement areas of I-ACT | Some parts of the I-ACT content could have been improved |
|  |  | The intervention procedure could have been improved |
|  |  | Exercises could work less well |
|  |  | Advantages of other formats |
|  | Procedure feasibility | Good that the sports psychologist reached out regularly |
|  |  | Positive with the chat function with the sports psychologist |
|  |  | It took a reasonable amount of time to fill out the questionnaires |
|  |  | An appropriate amount of content |
|  | The role of the context during an individual psychological intervention in sports | Partially involving the environment can be positive |
|  |  | The influence of the environment |
| The relevance of psychological skills | It was possible to put the skills into practice | Useful exercises for sports |
|  |  | Generalizable to different parts of sports training and other activities |
|  |  | I-ACT skills could be automated and informally practiced |
|  |  | ACT consistent behavior |
|  |  | Regular training is needed |
|  | I-ACT was helpful | Helpful for sports |
|  |  | Helpful for life outside of sports |
|  |  | More efficient behavioral patterns |
|  |  | Relevant for ice hockey players |
|  |  | I-ACT met the athlete's needs |
|  |  | Did not experience negative effects of I-ACT |
|  |  | I-ACT could be customized for individuals |
|  |  | I-ACT was appreciated |
