# Supplementary material for: Development and experiences of an internet-based acceptance and commitment training (I-ACT) intervention in ice hockey players: a qualitative feasibility study
Source: Front Sports Act Living. 2024 Mar 22;6:1297631. doi: 10.3389/fspor.2024.1297631 (PMC10995355; doi:10.3389/fspor.2024.1297631)
Supplement: Supplementary file 3 [file Table3.docx]

**Table 3.** I-ACT content ratings

|  | Module 1 | Module 2 | Module 3 | Module 4 | Module 5 | Module 6 | Module 7 | Summary of all 7 modules |
| --- | --- | --- | --- | --- | --- | --- | --- | --- |
| N completed the module | 4 | 4 | 4 | 4 | 4 | 3 | 2 |  |
| N completed the module rating | 4 | 3 | 4 | 4 | 4 | 3 | 2 |  |
| Total modules completed (1-7) | |  |  |  |  |  |  | 6.25 (0.96) |
| Did you understand the content of the module? (1-5) | 4.75 (0.5) | 4.33 (0.58) | 4.25 (0.96) | 4.5 (0.58) | 4.75 (0.5) | 5 (0) | 5 (0) | 4.62 (0.58) |
| Was the content of the module important for you as an ice hockey player? (1-5) | 4.75 (0.5) | 4.67 (0.58) | 4.25 (0.96) | 5 (0) | 4.75 (0.5) | 5 (0) | 5 (0) | 4.75 (0.53) |
| How easy was it to read the text? (1-5) | 4.5 (0.58) | 4.33 (0.58) | 4.5 (0.58) | 4.5 (0.58) | 4.5 (0.58) | 5 (0) | 5 (0) | 4.58 (0.5) |
| What did you think about the audio exercises? (1-5) | N/A | 3.67 (1.15) | 4.5 (0.58) | 4.25 (0.96) | 4.67 (0.58) | 5 (0) | 5 (0) | 4.47 (0.77) |
| What did you think about the videos? (1-5) | 4.75 (0.5) | 4 (1) | 4.75 (0.5) | 4.75 (0.5) | 4.67 (0.58) | N/A | 5 (0) | 4.65 (0.59) |
| What grade would give this module overall? | 4.5 (0.58) | 4.33 (0.58) | 4.5 (0.58) | 4.75 (0.5) | 4.5 (1) | 5 (0) | 5 (0) | 4.62 (0.58) |
| Would you recommend this module to other ice hockey players? (% Yes-answers) | 100% | 100% | 100% | 100% | 100% | 100% | 100% |  |
